# Molecular detection of fluoroquinolone-resistant *Neisseria meningitidis* by using mismatched PCR-restriction fragment length polymorphism technique

**DOI:** 10.3389/fcimb.2022.911911

**Published:** 2022-08-02

**Authors:** Yusuke Ota, Reina Okada, Hideyuki Takahashi, Ryoichi Saito

**Affiliations:** ^1^ Department of Molecular Microbiology, Graduate School of Medicine and Dental Science, Tokyo Medical and Dental University, Tokyo, Japan; ^2^ Department of Bacteriology I, National Institute of Infectious Diseases, Tokyo, Japan

**Keywords:** *gyrA*, Neisseria meningitidis, Acil, PCR-RFLP, fluoroquinolone resistance

## Abstract

Ciprofloxacin (CIP) is a commonly used antibiotic for meningococcal chemoprophylaxis, and the mutations in the quinolone resistance-determining region of *gyrA* are associated with CIP-resistant *Neisseria meningitidis*. Here, we established a mismatched PCR-restriction fragment length polymorphism (RFLP) assay to detect a mutation at codon 91 of *gyrA*, followed by high-level CIP-resistant meningococci. We designed PCR-RFLP primers to detect the T91I mutation in *gyrA* by introducing an artificial *Aci*I cleavage site. This assay was performed using 26 *N. meningitidis* strains whose *gyrA* sequences have been characterized. The amplified 160 bp PCR product from *gyrA* was digested into three fragments (80, 66, and 14 bp) when there was no mutation, or two fragments (146 and 14 bp) when there was a mutation at codon 91. A correlation was observed between the mismatched PCR-RFLP assay and *gyrA* sequencing. This rapid, simple, and accurate assay has the potential to detect CIP-resistant *N. meningitidis* in clinical microbiology laboratories, contributing to the appropriate antibiotic selection for meningococcal chemoprophylaxis, will help maintain an effective treatment for close contacts of IMD patients, and prevent the spread of CIP-resistant *N. meningitidis*.

## Introduction

*Neisseria meningitidis* is a major cause of life-threatening sepsis and meningitis. Globally, *N. meningitidis* is responsible for at least 1.2 million invasive meningococcal disease (IMD) cases, causing 135,000 deaths every year (Jafri et al., 2013). Therefore, adequate and quick management of IMD is necessary to control the spread of infection. Close contacts of patients are at an increased risk for IMD as the bacteria are easily transmitted from one person to another through respiratory or throat secretions (Cohn et al., 2013). According to the Center for Disease Control and Prevention recommendations, meningococcal chemoprophylaxis should be implemented for close contacts of patients with IMD to prevent its secondary cases, and ciprofloxacin (CIP) is one of the three antibiotics recommended for chemoprophylaxis (McNamara et al., 2018). It has been reported that CIP administration within 72 hours of case notification reduced the overall meningitis attack rate in a clinical trial of CIP chemoprophylaxis for contacts of IMD patients (Coldiron et al., 2018). However, several CIP-resistant *N. meningitidis* isolates have been reported worldwide (Shultz et al., 2000; Alcala et al., 2004; Corso et al., 2005; Mehta and Goyal, 2007; Singhal et al., 2007; Enriquez et al., 2008; Strahilevitz et al., 2008; Wu et al., 2009; Du Plessis et al., 2010; Bukovski et al., 2016; Tsang et al., 2017; Gorla et al., 2018; Kawasaki et al., 2018). There was a case reported in which CIP was administered for IMD close contacts before antibiotic susceptibility result was available, but CIP resistance was later confirmed and the patient was switched to the remaining agents in the chemoprophylaxis recommendation (Kawasaki et al., 2018). The hot-spot region of the *gyrA* in CIP-resistant meningococci is codon 91 in the quinolone resistance-determining region (QRDR), (Alcala et al., 2004; Singhal et al., 2007; Enriquez et al., 2008; Strahilevitz et al., 2008; Wu et al., 2009; Du Plessis et al., 2010; Hong et al., 2013; Chen et al., 2015; Bukovski et al., 2016; Tsang et al., 2017; Gorla et al., 2018; Kawasaki et al., 2018; Chen et al., 2020; McNamara et al., 2020; Zhao et al., 2020), thus a rapid and accurate diagnostic tool to detect such mutations is needed for maintaining efficacious treatment for close contacts of IMD in clinical microbiology laboratories.

Most clinical laboratories use culture-based phenotypic methods, such as disc diffusion and broth microdilution, to detect CIP-resistant isolates, as described by the guidelines of the Clinical and Laboratory Standards Institute (CLSI) (Clinical and Laboratory Standards Institute, 2022). Enriquez et al. also suggested that using the disc diffusion method with nalidixic acid may predict isolates with *gyrA* mutations that decrease the activity of fluoroquinolones (Enriquez et al., 2009). Another study reported that the T91I mutation in *gyrA* may be responsible for *in vivo* CIP resistance, indicating that a reliable screening method by sequencing *gyrA* is required (Hong et al., 2013). Although phenotypic or *gyrA* sequencing methods can detect CIP resistance, these methods are time-consuming, and require more than a day to complete from pure-cultured colonies. PCR-restriction fragment length polymorphism (RFLP) detects minor variations in a gene, where a single-base substitution creates or abolishes a recognition site for the restriction enzyme (Hashim and Al-Shuhaib, 2019). A PCR-RFLP-based rapid assay has been used to detect mutations linked to fluoroquinolone resistance in many species of bacteria (Alonso et al., 2004; Zhao et al., 2012; Nakano et al., 2013; Sierra-Arguello et al., 2018; Kakuta et al., 2020), but the assay for detecting *gyrA* mutations has not been reported for use in *N. meningitidis*.

In this study, we established a novel PCR-RFLP technique for the detection of *gyrA* mutations associated with high-level CIP-resistant *N. meningitidis*. This assay will contribute to adequate antibiotic selection for the prevention of secondary infection by IMD.

## Materials and methods

### Bacterial isolates

*Neisseria meningitidis* MC58 was obtained from the American Type Culture Collection (Manassas, VA, USA) and was used as a control strain with wild-type *gyrA*. We also used CIP-susceptible *N. gonorrhoeae* clinical isolate and six major causative organisms of meningitis (*Escherichia coli* ATCC 25922, *Pseudomonas aeruginosa* ATCC 27853, *Haemophilus influenzae* ATCC 49247, *Streptococcus pneumoniae* ATCC 49619, *Streptococcus agalactiae* clinical strain, and *Listeria monocytogenes* clinical strain) to estimate the specificity of our method. We analyzed all available CIP-intermediate (n = 5) and CIP-resistant (n = 6) *N. meningitidis* isolates and randomly selected CIP-susceptible *N. meningitidis* strains (n = 14) obtained between 1998 and 2018 at the National Institute of Infectious Diseases, Japan. These 25 non-duplicate *N. meningitidis* strains were isolated from clinical specimens (17 sterile and 8 non-sterile samples). Each strain was inoculated on chocolate agar plates and incubated at 37°C with 5% CO_2_. Ciprofloxacin susceptibility was previously determined using the E-test strip (bioMérieux, Marcy IEtoile, France) (Saito et al., 2022; Clinical and Laboratory Standards Institute, 2022) (**Table 1**). Single nucleotide polymorphisms within the QRDR of *gyrA* were determined by sequencing analysis using specific primers (Wu et al., 2009).

**Table 1:** Distribution of CIP susceptibility, GyrA substitution, and *Aci*I digestion pattern in meningococcal strains.

Strain	CIP susceptibility (μg/mL)	GyrA substitution	*Aci*I digestion	Accession number
MC58	0.003 (S)	–	+	AE002098.2
NIID416	≦0.002 (S)	–	+	ON382529
NIID536	≦0.002 (S)	–	+	ON382535
NIID287	0.003 (S)	–	+	ON382523
NIID289	0.003 (S)	–	+	ON382524
NIID345	0.003 (S)	–	+	ON382525
NIID358	0.003 (S)	–	+	ON382526
NIID411	0.003 (S)	–	+	ON382528
NIID418	0.003 (S)	–	+	ON382531
NIID471	0.003 (S)	–	+	ON382532
NIID507	0.003 (S)	–	+	ON382533
NIID560	0.003 (S)	–	+	ON382536
NIID599	0.003 (S)	–	+	ON382539
NIID375	0.004 (S)	–	+	ON382527
NIID535	0.008 (S)	–	+	ON382534
NIID584	0.064 (I)	D95Y (G283T)	+	ON382538
NIID624	0.064 (I)	D95Y (G283T)	+	ON382543
NIID699	0.064 (I)	D95Y (G283T)	+	ON382546
NIID620	0.094 (I)	T91I (C272T, C273T)	–	ON382542
NIID727	0.094 (I)	D95Y (G283T)	+	ON382547
NIID417	0.125 (R)	T91I (C272T, C273T)	–	ON382530
NIID576	0.125 (R)	T91I (C272T, C273T)	–	ON382537
NIID614	0.125 (R)	T91I (C272T, C273T)	–	ON382541
NIID600	0.190 (R)	T91I (C272T, C273T)	–	ON382540
NIID670	0.190 (R)	T91I (C272T, C273T)	–	ON382545
NIID652	0.250 (R)	T91I (C272T)	–	ON382544

S, susceptible; I, intermediate; R, resistant.

### Development of the mismatched PCR-RFLP assay

**Figure 1:**
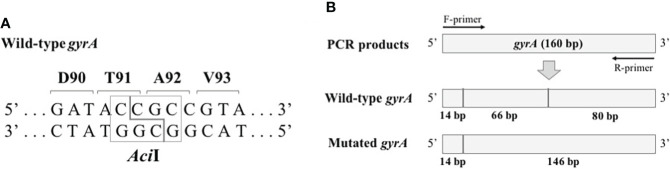
Schematic representation of the mismatched PCR-RFLP assay. **(A)** *Aci*I recognition in isolates with wild-type *gyrA*. **(B)** The predicted fragment pattern after *Aci*I digestion. The vertical line represents the *Aci*I recognition site.

Based on the DNA sequence of *gyrA* of *N. meningitidis* MC58 (GenBank accession number: AE002098.2) and clinical *N. meningitidis* isolates, we designed PCR-RFLP primers to detect the T91I mutation in *gyrA* by introducing an artificial *Aci*I (CCGC) (New England Biolabs, MA, USA) cleavage site into the PCR products, generating DNA fragments of sizes that may be identified by electrophoresis (**Figure 1A**). The nucleotides were used in the forward primer NM_gyrA_AciI-F1 (5′- AACAACTGGAATK**CCGC**CTACA-3′) and reverse primer NM_gyrA_AciI-R2 (5′-CGAAGTTGCCYTGWCCGTC-3′). The primer sequences and PCR conditions were expected to yield 160 bp DNA fragments for *gyrA*. The amplified PCR product was digested with *Aci*I restriction enzyme, resulting in 80, 66, and 14 bp fragments in isolates with wild-type *gyrA*, and 146 and 14 bp fragments in isolates with a T91I mutation in *gyrA* (**Figure 1B**).

### Determining the *gyrA* mutation using mismatched PCR-RFLP assay

The genomic DNA of each bacterial isolate was extracted from the overnight culture on the agar plate using NucleoSpin^®^ Tissue (TaKaRa, Shiga, Japan) according to the manufacturer’s instructions. The PCR was carried out in a 20 μL reaction mixture containing 10 μL of EmeraldAmp MAX PCR Master Mix (TaKaRa), 1 μL (10 pmol) of each primer, 7 μL of deionized distilled water, and 1 μL of extracted template DNA. Amplification was performed using an Applied Biosystems thermal cycler (Foster City, CA, USA) under the following amplification conditions: 30 cycles of denaturation at 98 °C for 10 s, annealing at 55 °C for 30 s, and extension at 72 °C for 1 min. The PCR products were digested with *Aci*I. The reaction mixture contained 5 μL of PCR product, 1 μL of *Aci*I, 1 μL of CutSmart buffer (New England Biolabs), and 3 μL of deionized distilled water and was carried out at 37 °C for 10 min. The digested DNA fragments were electrophoresed on a 3.0% agarose gel (PrimeGel ™ Agarose LE 1-20K, TaKaRa) and band patterns were visualized using a UV transilluminator (ATTO, Tokyo, Japan) after ethidium bromide staining.

## Results

### Mutations in QRDR of *gyrA*

The DNA sequence analysis showed that all CIP-intermediate and -resistant strains had mutations in QRDR of the *gyrA* (**Table 1**). Seven strains showed T91I caused by point mutations of C272T and C273T or a mutation of C272T. Four strains indicated a point mutation at codon 95 (GAC→TAC), leading to the replacement of Asp by Tyr. These mutations were not detected in any of the remaining 14 isolates. The *Aci*I recognition site was abolished only by the mutations of T91I.

### Mismatched PCR-RFLP assay

**Figure 2:**
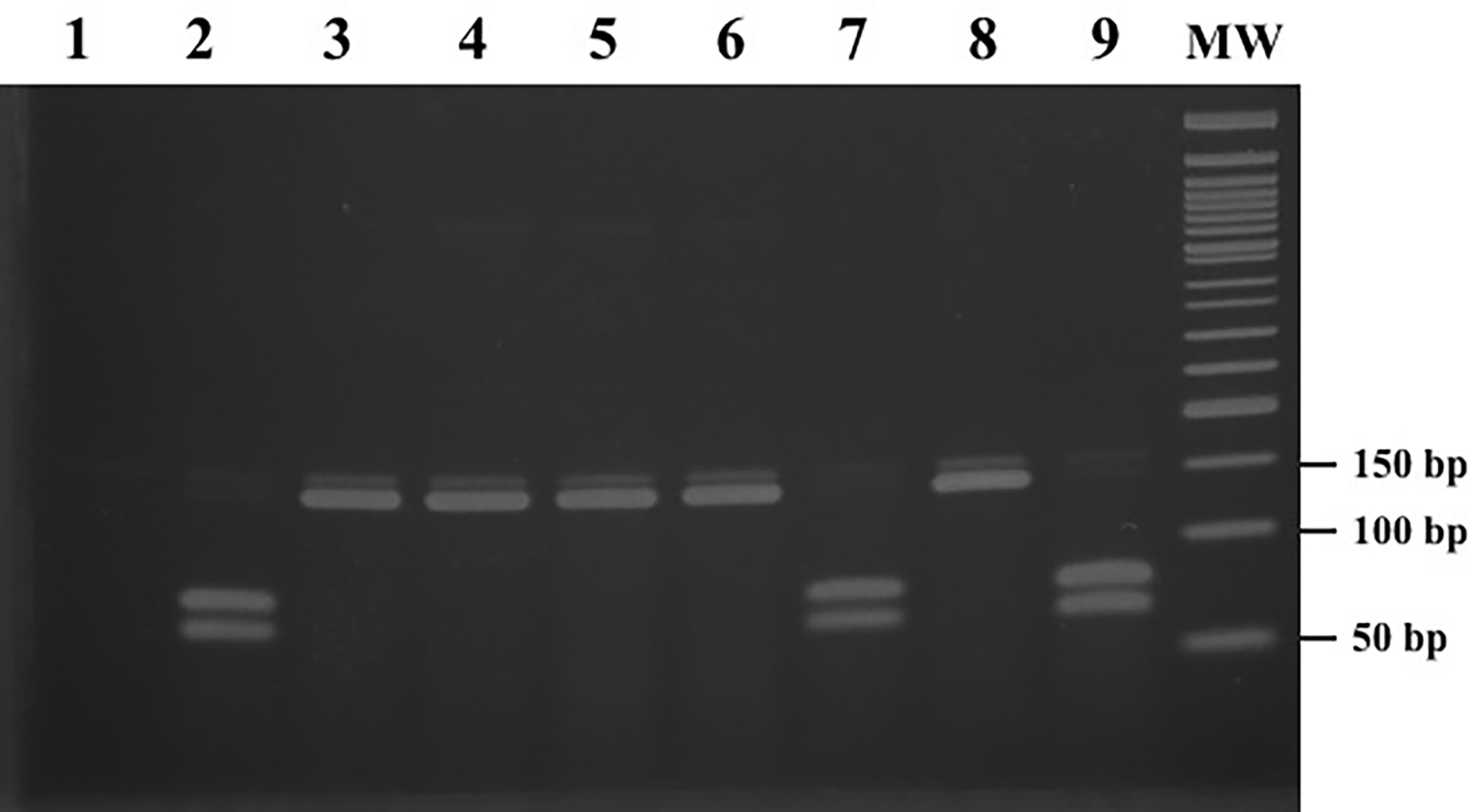
PCR-RFLP patterns obtained after digestion with *Aci*I for *gyrA*. Lane 1: negative control; lanes 2, 7, and 9: isolates with wild-type *gyrA*; lanes 3-6: 91 codon ACC → ATT; lane 8: 91 codon ACC → ATC; lane MW: 50 bp ladder molecular-mass standard.

The results of the mismatched PCR-RFLP assay for *gyrA* are shown in **Figure 2** and **Table 1**. The PCR amplification products with an expected size of 160 bp for *gyrA* were successfully obtained for all *N. meningitidis* strains. When the amplicon was digested with *Aci*I, isolates with wild-type *gyrA* showed two fragments of size 80 and 66 bp. However, following digestion with *AciI*, isolates that had a T91I mutation in *gyrA* produced a 146 bp fragment. The 14 bp fragment produced following *Aci*I digestion was not visible in any of the meningococcal isolates. A correlation was observed between the mismatched PCR-RFLP assay and *gyrA* sequencing.

Comparing the results to CIP susceptibility, all 15 CIP-susceptible strains with wild-type *gyrA* showed positive *Aci*I digestion results. All 6 CIP-resistant strains carrying a T91I mutation in *gyrA* indicated a negative *Aci*I digestion pattern. Four of the five CIP-intermediate strains with D95Y in *gyrA* exhibited positive *Aci*I digestion results, while the remaining one strain which had a T91I mutation in *gyrA* showed a negative result.

The DNA of major meningitis-causing organisms other than *N. meningitidis* was not amplified by the primers used in this study (**Supplementary Figure 1**). Besides, CIP-susceptible *N. gonorrhoeae* showed a PCR product of about 160 bp by PCR amplification, and the product was digested with *Aci*I, leading to possessing wild-type *gyrA* (**Supplementary Figure 1**).

## Discussion

Administration of appropriate antibiotics in the control of IMD as well as prompt assay to detect antimicrobial resistance in a microbiology laboratory are essential. In the present study, we describe a novel mismatched PCR-RFLP assay for detecting CIP-resistant *N. meningitidis*. Our results indicate that the assay detects a mutation in codon 91 of *gyrA*, followed by high-level CIP resistance with 100% sensitivity and specificity in meningococci. Furthermore, this assay provides results within 4 h, which is much faster than conventional methods. In a previous study that evaluated the utility of rapid susceptibility testing in bloodstream infections caused by gram-negative bacteria, the time to effective antibiotic was shorter in the rapid testing group (Anton-Vazquez et al., 2022). Therefore, our accurate and rapid assay could be useful for adequate antibiotic selection in clinical microbiology laboratories.

In this assay, the restriction enzyme *Aci*I recognizes and digests the last two nucleotides of codon 91 and the first two nucleotides of codon 92 of *gyrA*, in isolates with no mutations in this region. In contrast, isolates with the second nucleotide substitution (C272T) of codon 91 of *gyrA*, resulting in the T91I mutation, exhibit an undigested pattern. Thus, the assay can detect the T91I mutation leading to CIP resistance in our isolates with high accuracy. However, the predicted 14 bp band was not visible in our results. This fragment related *Aci*I recognition site on the forward primer is not directly involved in the T91 mutation, suggesting that the 14 bp fragment does not affect CIP resistance. Although no PCR amplified bands were observed in major meningitis-causing organisms, CIP-susceptible *N. gonorrhoeae* showed a PCR product using our primer set, suggesting that our PCR primers are not specific to *N. meningitidis*.

The number of resistant pathogens increases every year, mainly because of antibiotic misuse (Aslam et al., 2018). Therefore, the administration of adequate antibiotics against the target microbes and prompt antimicrobial resistance testing are essential (Frickmann et al., 2014). Ciprofloxacin and rifampicin are the primary antibiotics recommended for oral chemoprophylaxis of meningococcal disease for close contacts of IMD patients (McNamara et al., 2018). Lodi et al. reported the case of an IMD with rifampicin resistance secondary to chemoprophylaxis, resulting in limited antibiotic options available for the IMD case (Lodi et al., 2020). Although meningococcal resistance to CIP is uncommon, CIP-resistant isolates have emerged worldwide, which is a serious concern for chemoprophylaxis failure (Shultz et al., 2000; Alcala et al., 2004; Corso et al., 2005; Mehta and Goyal, 2007; Singhal et al., 2007; Enriquez et al., 2008; Strahilevitz et al., 2008; Wu et al., 2009; Du Plessis et al., 2010; Bukovski et al., 2016; Tsang et al., 2017; Gorla et al., 2018; Kawasaki et al., 2018). Quinolone and its derivatives, such as CIP, inhibit the action of DNA gyrase (GyrA/GyrB) and topoisomerase IV (ParC/ParE). Amino acid substitutions in these enzymes are involved in the development of quinolone resistance (Ruiz, 2003). In a previous study, mutations within the QRDRs *gyrA*, *gyrB*, *parC*, and *parE* were analyzed to characterize fluoroquinolone resistance mechanisms in *N. meningitidis* (Corso et al., 2005; Chen et al., 2015; Tsang et al., 2017; Kawasaki et al., 2018; Chen et al., 2020). Meningococcal CIP resistance is mainly due to point mutations in the QRDR of *gyrA*, but no mutations in the QRDRs of *gyrB*, *parC*, and *parE* have been found (Corso et al., 2005; Chen et al., 2015; Tsang et al., 2017). Although few studies have reported that CIP-resistant *N. meningitidis* isolates have point mutations in *gyrB*, *parC*, and *parE*, the isolates also showed T91I mutations in *gyrA* (Kawasaki et al., 2018; Chen et al., 2020). Collectively, we suggest that our mismatched PCR-RFLP assay, which can identify significant T91I mutations in *gyrA*, has high sensitivity for the detection of CIP-resistant meningococci.

The gold standard method for identifying gene mutations is direct DNA sequencing, and many studies have used this approach to detect *gyrA* mutations affecting fluoroquinolone-resistant *N. meningitidis* (Alcala et al., 2004; Singhal et al., 2007; Enriquez et al., 2008; Strahilevitz et al., 2008; Wu et al., 2009; Du Plessis et al., 2010; Hong et al., 2013; Chen et al., 2015; Bukovski et al., 2016; Tsang et al., 2017; Gorla et al., 2018; Kawasaki et al., 2018; Chen et al., 2020; McNamara et al., 2020; Zhao et al., 2020). Sequencing of DNA is the most reliable technique for determining nucleotide mutations; however, its routine use requires specialized instruments and extensive training of laboratory staff. The simple and cost-effective PCR-RFLP assay, which works based on the presence or absence of recognition sequences, offers an alternative means to characterize single nucleotide polymorphisms (Hashim and Al-Shuhaib, 2019) and is used to assess antimicrobial resistance among bacterial isolates (Alonso et al., 2004; Zhao et al., 2012; Nakano et al., 2013; Sierra-Arguello et al., 2018; Kakuta et al., 2020). We developed a PCR-RFLP methodology to detect single nucleotide substitutions associated with CIP-resistant meningococci. This simplification of measurement is important for practical use in the microbiology laboratory as a new testing method (Maurer et al., 2017). The other studies with a related species, *N. gonorrhoeae*, reported that detection of antibiotic resistance as point-of-care testing by a simple assay may extend the usefulness of existing antibiotics for treatment (Tuite et al., 2017; Turner et al., 2017). An integrated PCR system, which performs automated sample preparation and fast PCR, has been developed for application in point-of-care testing, suggesting that we might be able to further reduce the operation time of our PCR-RFLP assay by taking advantage of such a technique (Lee et al., 2021).

The increasing number of cases of CIP-resistant *N. meningitidis* demonstrate that reduced CIP susceptibility can be developed in *N. meningitidis* populations (Potts et al., 2021; Saito et al., 2022). In Japan, the geometric mean MICs tended to increase for CIP every seven years, due to the increasing rate of CIP-intermediate and -resistant isolates between 2012 and 2018 (Saito et al., 2022). Moreover, 11 penicillin- and CIP-resistant isolates containing *bla*_ROB-1_ and a *gyrA* T91I mutation were identified in the USA after December 2018 (Potts et al., 2021). These epidemiological data suggest the need for a wider understanding of the problems of developing the CIP-resistant *N. meningitidis* population using simple assays available in resource-limited laboratories and areas and our method can contribute to obtain detailed epidemiological information worldwide.

A limitation of this study is that the proposed method cannot detect mutations at other *gyrA* locations. Residue D95 mutation of *gyrA* has also been reported to be associated with decreased susceptibility to CIP (Shultz et al., 2000). Our four meningococcal isolates with this mutation showed intermediate level of CIP resistance. Other studies have also reported that the D95 mutation of *gyrA* lead to an increase in minimum inhibitory concentration to an intermediate level in *N. meningitidis* (Corso et al., 2005; Chen et al., 2015). Therefore, these data suggest that our assay is a useful tool for detecting highly CIP-resistant *N. meningitidis* isolates, and some low-level CIP-resistant isolates. However, CIP-resistant *N. meningitidis* isolates without the T91I mutation of *gyrA*, which may have multiple mutations in QRDRs, have been reported (Shultz et al., 2000; Enriquez et al., 2008; Strahilevitz et al., 2008). It is necessary to further optimize this method to detect other *gyrA* mutations in future studies.

In conclusion, we established a mismatched PCR-RFLP assay for detecting a mutation in codon 91 of *gyrA*, that is associated with high-level CIP-resistant *N. meningitidis*. A prompt method to detect CIP resistance is in high demand to prevent secondary cases of IMD. This rapid, simple, and accurate assay has the potential to be used daily for the detection of CIP-resistant meningococci in clinical microbiological laboratories, contributing to maintaining effective management of IMD cases for infection control and preventing the spread of CIP-resistant *N. meningitidis*.

## Data availability statement

The datasets presented in this study can be found in online repositories. The names of the repository/repositories and accession number(s) can be found in the article/**Supplementary Material**.

## Author contributions

RS designed, organized, and coordinated this project. YO was the chief investigator and responsible for the data analysis. RO contributed to the acquisition of the data. HT contributed to the interpretation of the data. YO and RS wrote the initial and final drafts of the manuscript. All authors revised the drafts of the manuscript and approved the final version of the manuscript.

## Funding

This work was supported by the Japan Agency for Medical Research and Development (AMED) under Grant Number JP20fk0108071 (HT, RS), and the JSPS KAKENHI Grant Number JP20K08818 (RS). These funders had no role in the study design, data collection and analysis, decision to publish, or preparation of the manuscript.

## Acknowledgments

We would like to thank Editage (www.editage.com) for English language editing.

## Conflict of interest

The authors declare that the research was conducted in the absence of any commercial or financial relationships that could be construed as a potential conflict of interest.

## Publisher’s note

All claims expressed in this article are solely those of the authors and do not necessarily represent those of their affiliated organizations, or those of the publisher, the editors and the reviewers. Any product that may be evaluated in this article, or claim that may be made by its manufacturer, is not guaranteed or endorsed by the publisher.

## Abbreviations

CLSI, Clinical and Laboratory Standards Institute; CIP, ciprofloxacin; IMD, invasive meningococcal disease; QRDR, quinolone resistance determining region; RFLP, restriction fragment length polymorphism.
