# Supplementary material for: Molecular detection of fluoroquinolone-resistant Neisseria meningitidis by using mismatched PCR-restriction fragment length polymorphism technique
Source: Front Cell Infect Microbiol. 2022 Aug 2;12:911911. doi: 10.3389/fcimb.2022.911911 (PMC9378782; doi:10.3389/fcimb.2022.911911)
Supplement: Supplementary file 1 [file DataSheet_1.zip › Supplementary/Supplementary_Figure_legend.docx]

**Supplementary Figure Legend.**

**Supplementary Figure 1. PCR and PCR-RFLP patterns in strains other than *Neisseria meningitidis*.** Lane 1: negative control; lane 2: ciprofloxacin (CIP)-susceptible *N. meningitidis* MC58 with wild-type *gyrA*; lane 3: CIP-susceptible *Neisseria gonorrhoeae* clinical strain; lane 4: *Escherichia coli* ATCC 25922; lane 5: *Pseudomonas aeruginosa* ATCC 27853; lane 6: *Haemophilus influenzae* ATCC 49247; lane 7: *Streptococcus pneumoniae* ATCC 49619; lane 8: *Streptococcus agalactiae* clinical strain; lane 9: *Listeria monocytogenes* clinical strain; lane 10: CIP-susceptible *N. meningitidis* MC58 with wild-type *gyrA* after *Aci*I digestion; lane 11: CIP-susceptible *N. gonorrhoeae* clinical strain after *Aci*I digestion; lane MW: 50 bp ladder molecular-mass standard.
