# Supplementary figures and images for: Molecular detection of fluoroquinolone-resistant Neisseria meningitidis by using mismatched PCR-restriction fragment length polymorphism technique
Source: Front Cell Infect Microbiol. 2022 Aug 2;12:911911. doi: 10.3389/fcimb.2022.911911 (PMC9378782; doi:10.3389/fcimb.2022.911911)

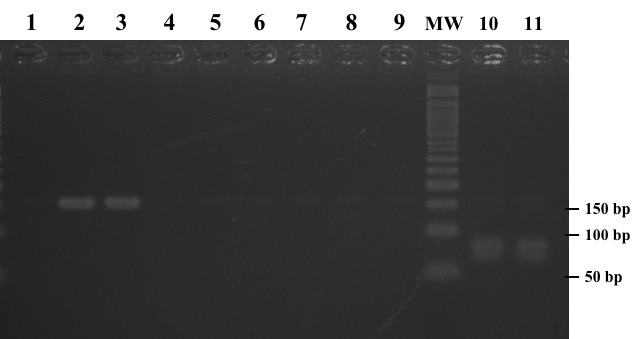

Supplement: Supplementary file 1 [file DataSheet_1.zip › Supplementary/Supplementary Figure 1.jpg]
